# EXPLORING THE EFFECTS OF AN EIGHT-WEEK LIFESTYLE INTERVENTION ON REST-ACTIVITY RHYTHM IN ADULTS

**DOI:** 10.1093/geroni/igae098.3522

**Published:** 2024-12-31

**Authors:** Meina Zhang, Michelle Voss, Lucas Carr, Kara Whitaker, Jenna Springer, Chooza Moon

**Affiliations:** University of Iowa, Iowa City, Iowa, United States; University of Iowa, Iowa City, Iowa, United States; University of Iowa, Iowa City, Iowa, United States; University of Iowa, Iowa City, Iowa, United States; University of Iowa, Iowa City, Iowa, United States; University of Iowa, Iowa City, Iowa, United States

## Abstract

Rest activity rhythm (RAR) describes the pattern of physical activity and sleep in 24 hours. Disruptions in RAR have been associated with various age-related chronic conditions, including sleep disorders, depression, and increased risk for cognitive decline. Few studies examined if RAR changes from intervention that includes both physical activity and sleep components. This study investigated the effects of an 8-week lifestyle intervention targeting the full 24-hour activity profile (physical activity, sedentary behavior, and sleep) on RAR. Participants engaged in up to five sessions with a student health coach, viewed three health education videos targeting physical activity, sedentary behavior, and sleep, and received an Acttrust2 activity monitor. A cohort of 26 participants (mean age = 49.3 years, SD = 11.1; 73.1% female) was monitored using wrist-worn Acttrust2 devices for one week pre- and post-intervention to observe changes in RAR. RAR parameters were analyzed using cosinor approach, such as amplitude (maximum activity level), mesor (mean activity level), pseudo-F-statistic (the robustness of RAR), and acrophase (peak activity time). The intervention showed mixed effects on RAR parameters. While not statistically significant, positive changes were observed in amplitude (Cohen’s d=0.24) and mesor (Cohen’s d=0.12). Conversely, the pseudo-F-statistic (Cohen’s d = -0.002) and acrophase (Cohen’s d=-0.02) remained stable post-intervention. Although statistically significant changes in RAR parameters were not observed, encouraging trends suggest the potential for the intervention to influence maximum and mean physical activity level. These findings contribute to our understanding of lifestyle interventions in adults and highlight areas for further research and intervention refinement.

